# Prognostic value of stress CMR and SPECT-MPI in patients undergoing intermediate-to-high-risk non-cardiac surgery

**DOI:** 10.1007/s11547-024-01876-x

**Published:** 2024-09-10

**Authors:** Fabio Fazzari, Costanza Lisi, Federica Catapano, Francesco Cannata, Federica Brilli, Stefano Figliozzi, Renato Maria Bragato, Giulio Giuseppe Stefanini, Lorenzo Monti, Marco Francone

**Affiliations:** 1 IRCCS Humanitas Research Hospital, Via Manzoni 56, 20089 Rozzano, Milan, Italy; 2 Department of Biomedical Sciences, Humanitas University, Via Rita Levi Montalcini 4, 20090 Pieve Emanuele, Milan, Italy; 3 Department of Perioperative Cardiology and Cardiovascular Imaging, Centro Cardiologico Monzino IRCCS, Milan, Italy

**Keywords:** Stress cardiac magnetic resonance, SPECT-MPI, Non-cardiac surgery, Risk stratification, Cardiovascular complications

## Abstract

**Purpose:**

The objective of this study was to investigate the role of myocardial perfusion imaging (MPI) stress tests using stress cardiac magnetic resonance (sCMR) and single-photon emission computed tomography myocardial perfusion imaging (SPECT-MPI) in non-cardiac surgery (NCS) pre-operatory management.

**Materials and methods:**

This monocentric retrospective study enrolled patients with coronary artery disease or a minimum of two cardiovascular risk factors undergoing intermediate-to-high-risk non-cardiac surgeries. The primary composite endpoint comprised cardiac death, cardiogenic shock, acute coronary syndromes (ACS), and cardiogenic pulmonary edema occurring within 30 days after surgery, while the secondary endpoint was ACS.

**Results:**

A total of 1590 patients were enrolled; among them, 669 underwent a MPI stress test strategy (sCMR: 287, SPECT-MPI: 382). The incidence of 30-day cardiac events was lower in the stress-tested group compared to the non-stress-tested group (1.2% vs. 3.4%; *p* 0.006). Adopting a stress test strategy showed a significant reduction in the risk of the composite endpoint (OR: 0.33, 95% CI: 0.15–0.76, *p* 0.009) and ACS (OR: 0.41, 95% CI: 0.17–0.98, *p* 0.046) at multivariable analysis, with similar cardiac events rate between stress CMR and SPECT (1.1% vs. 1.3%, *p* 0.756). Stress CMR showed a greater accuracy to predict coronary artery revascularizations (sCMR c-statistic: 0.95, ischemic cut-point: 5.5%; SPECT c-statistic: 0.85, ischemic cut-point: 7.5%).

**Conclusion:**

Stress test strategy is related to a lower occurrence of cardiac events in high-risk patients scheduled for intermediate-to-high-risk non-cardiac surgeries. Both sCMR and SPECT-MPI comparably reduce the likelihood of cardiac complications, albeit sCMR offers greater accuracy in predicting coronary artery revascularization.

**Supplementary Information:**

The online version contains supplementary material available at 10.1007/s11547-024-01876-x.

## Introduction

Perioperative cardiac complications among patients undergoing non-cardiac surgery (NCS) range from less than 1% to 5%, depending on the type of intervention, patient risk profile, and preoperative cardiac risk assessment strategy. In patients with known coronary artery disease (CAD) or at least two cardiovascular risk factors and poor functional capacity, an imaging stress test allows to detect inducible myocardial ischemia and should call for specific risk reduction strategies before intermediate-to-high-risk NCS [1]. However, data supporting cardiac imaging modalities in patients undergoing NCS are sparse and out of date [2–5]. In particular, there is little specific evidence on the use of stress cardiac magnetic resonance (sCMR) in this setting, even if this myocardial perfusion imaging (MPI) modality is widely used nowadays for myocardial ischemia detection in patients with known or suspected CAD[6, 7]. Moreover, modality-specific thresholds of inducible ischemia associated with an increased perioperative risk are poorly defined. As a result, there is no consensus on the best strategy for risk stratification in this setting. The aim of the present study is to assess the prognostic role of a myocardial perfusion imaging (MPI) stress test strategy based on sCMR or stress single-photon emission computed tomography myocardial perfusion imaging (SPECT-MPI) in terms of 30-day post-surgery cardiac events.

## Methods

**Fig. 1:**
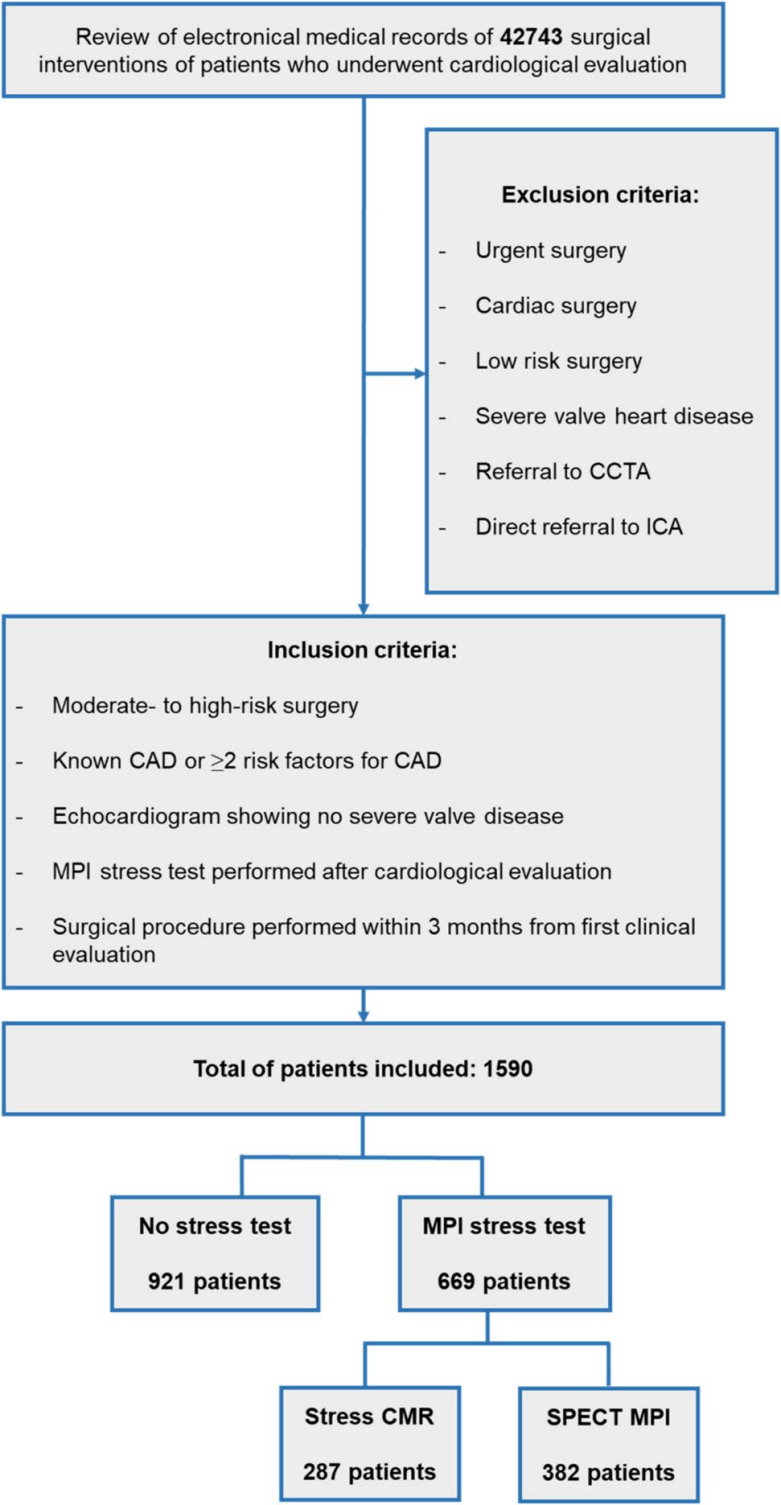
Study workflow. *CAD* coronary artery disease, *CCTA* coronary computed tomography angiography, *ICA* invasive coronary angiography, *MPI* myocardial perfusion imaging, *sCMR* stress cardiac magnetic resonance, *SPECT-MPI *single-photon emission computed tomography myocardial perfusion imaging

This is a single-center, retrospective cohort study conducted at a tertiary academic hospital. The study protocol was approved by the institutional ethics committee, and given the retrospective design of the study, the ethics committee waved the need of specific informed consent. Consecutive patients undergoing NCS between January 2015 and December 2021 were scrutinized and included according to prespecified inclusion and exclusion criteria (Fig. 1). Patients candidate to urgent, cardiac, and low-risk surgery were excluded, as well as those affected by severe valvular disease. Referral to CCTA after the preoperative cardiologic evaluation or direct referral to ICA was considered exclusion criteria.

Electronic medical records were retrospectively reviewed to collect clinical and outcomes data. Data of interest included symptoms at the time of first evaluation, laboratory test values, reports of ECG, echocardiogram, sCMR and SPECT-MPI, ICA, and cardiovascular outcomes at 30-day post-surgery follow-up. Moderate–severe chronic kidney disease was defined as a glomerular filtration rate inferior to 45 ml/min/1.73 m^2^ according to CKD-EPI formula. Functional capacity was expressed in metabolic equivalents (METS) and reported during cardiological evaluation by asking the patient about daily activities (MET-REPAIR or DASI questionnaires were alternatively used [8]). Different types of surgeries were classified according to ESC guidelines in intermediate and high risk; revised cardiac risk index (RCRI) was computed through analysis of electronic medical records and the scores were assessed as suggested by guidelines[1]. The American Society of Anesthesiologists (ASA) class was evaluated for each patient [9]. The referral for stress testing was guided by real life clinical practice, as the result of multidisciplinary integration of patient clinical presentation, risk stratification, and functional capacity (revised cardiac risk index score, METS). To address potential referral/selection bias, we performed a propensity score matching analysis.

### Myocardial perfusion imaging stress test

Stress CMR was performed using adenosine (infusion rate 148 mcg/kg/min) or regadenoson (400 mcg i.v. bolus) with a rest stress protocol [10]. A Philips Achieva 1,5 T MRI system was used from 2015 to 2018; after this period, it was replaced by a Siemens Magnetom Aera 1,5 T MRI. Interpretation of CMR images and stress perfusion defects was made according to the recommendations of Society for Cardiovascular Magnetic Resonance [11]. Inducible perfusion defects were defined as an area of hypointensity signal that involves at least one myocardial segment with coronary distribution and persists for at least 3 phases after peak of contrast enhancement without evidence of LGE in the same position. A model of 17 segments was used to assess the number of ischemic segments and the percentage of ischemia (6% for each segment: 3% if less than 50% wall thickness and 6% in more than 50% wall thickness). CMR analysis was performed using Circle Cardiovascular Imaging software (cvi42, Calgary, Canada).

SPECT-MPI was conducted following a stress rest protocol; the choice of the type of stressor (physical, adenosine, regadenoson) was based on patients’ clinical characteristics. A Symbia Intevo™ Excel SPECT/CT Siemens System was used. All tests were performed using Technetium-99 m sestamibi. Acquisition protocol and images analysis followed the guidelines of the American Society of Nuclear Cardiology [12]. SPECT images were analyzed using standard 17-segment model. A grading scale from 0 to 4 was used to assess the severity of reduction in tracer uptake. Philips IntelliSpace Portal software was used for SPECT images postprocessing.

### Definition of clinical outcome and follow-up.

The prespecified primary outcome was a composite endpoint of cardiovascular complications during the 30-day period after surgery, including cardiac death, cardiogenic shock, acute coronary syndromes (ACS), and cardiogenic pulmonary edema. Secondary endpoint was ACS (defined as myocardial infarction and unstable angina).

## Statistical analysis

Descriptive statistics were obtained for all study variables. Continuous variables were presented as mean ± standard deviation (SD) or as median value and interquartile range (IQR), while categorical variables were presented as absolute number and percentage. Normality of data distribution was verified using the Shapiro–Wilk test. Comparisons between continuous variables were tested using the unpaired t-test or the Mann–Whitney test, as indicated. Relationships between categorical variables were tested using the Chi-square test or the Fisher’s exact test, as appropriate. Multivariable logistic regression analyses were performed to identify independent predictors of outcomes (primary composite endpoint and acute coronary syndromes) in the overall population and independent predictors of myocardial ischemia in the stress-tested group. Only variables that were significantly associated with the endpoint at univariable analysis (*p* < 0.05) were included in multiple multivariable logistic regression analyses. Results were expressed as odds ratio (OR) and confidence intervals (CI). Receiver operating characteristic (ROC) area under the curve (AUC), 95%CI, and the optimal cutoff points were calculated with Youden method to assess the predictive discriminatory power for coronary artery revascularization of percentage myocardial ischemia for both sCMR and SPECT-MPI. To examine the effect of imaging stress test in different subgroups, a test for interaction was performed; *p* for interaction was considered significant if < 0.05. Propensity score was calculated from a logistic regression model that included all baseline characteristics. Matching was done using a 1:1 nearest neighbor matching algorithm without replacement, with a caliper width of 0.2 of the standard deviation of the logit of the propensity score. To improve the discriminative capability of our models, we calculated the C-index for each model. The C-index, being numerically identical to the AUC of the ROC curve, provides an estimate of the model's discriminative power concerning the outcomes. We used the DeLong test to compare the AUCs of the ROC curves between different models. Statistical analysis was performed with Stata version 17.0 (StataCorp).

## Results

### Characteristics of the patient population

After the screening of 42743 surgical interventions, 1590 patients (42%) were finally included. Baseline clinical characteristics and outcomes of the study population, stratified according to the use of stress test modality, are shown in Table 1. Patients who underwent SPECT-MPI stress strategy showed higher ASA class and more often reported the assumption of cardioprotective therapies (i.e., anti-platelets and lipid-lowering drugs).

**Table 1:** Clinical characteristics of the study population divided in two groups (no stress test vs. MPI stress test). Values are indicated as number (n) and percentage (%), or median (interquartile range)

	Overall (1590 patients)	No stress test (921 patients)	MPI stress test (669 patients)	*p* value
*Demographic and clinical characteristics*				
Age	71 (65–77)	72 (65–77)	71 (64–77)	0.064
Female, n(%)	617 (38.8)	364 (39.5)	253 (37.8)	0.491
Familiar history of CAD, n(%)	295 (18.6)	172 (18.7)	123 (18.3)	0.883
Hypertension, n(%)	1290 (81.1)	755 (82.0)	535 (80.0)	0.313
Dyslipidemia, n(%)	1100 (69.2)	641 (69.6)	459 (68.6)	0.674
Diabetes, n(%)	520 (32.7)	304 (33.0)	216 (31.6)	0.762
Current or former smoking, n(%)	968 (60.8)	563 (61.2)	451 (67.4)	0.896
Coronary artery disease, n(%)	695 (43.7)	385 (41.8)	310 (46.3)	0.072
Prior myocardial infarction, n(%)	466 (29.3)	262 (28.4)	204 (30.5)	0.376
Prior PCI, n(%)	521 (32.7)	288 (31.2)	233 (34.8)	0.136
Prior CABG, n(%)	151 (9.5)	79 (8.6)	72 (10.8)	0.143
Atrial fibrillation, n(%)	177 (11.1)	93 (10.1)	84 (12.6)	0.114
Moderate–severe CKD, n(%)	260 (16.3)	141 (15.3)	120 (17.9)	0.145
Predicted METS range 4–10	1324 (83.3)	735 (79.8)	589 (88.0)	** < 0.001**
Predicted METS < 4	165 (10.4)	107 (11.6)	58 (8.7)	0.066
NYHA class II, n(%)	601 (38.0)	333 (36.2)	268 (40.0)	0.412
NYHA class III, n(%)	64 (4.0)	40 (4.3)	24 (3.6)	0.412
High-risk procedure, n(%)	805 (50.1)	443 (48.0)	362 (54.0)	**0.019**
Revised cardiac risk index score = 2, n(%)	631 (39.6)	351 (38.1)	280 (48.9)	0.133
Revised cardiac risk index score ≥ 3, n(%)	366 (23.0)	212 (23.0)	154 (23.0)	1.000
ASA class III, n(%)	772 (48.5)	411 (44.6)	361 (53.9)	** < 0.001**
ASA class IV, n(%)	51 (3.2)	31 (3.7)	20 (2.9)	0.774
*Symptoms*				
Typical angina, n(%)	67 (4.2)	5 (0.4)	62 (9.3)	** < 0.001**
Atypical angina, n(%)	426 (26.8)	250 (27)	176 (26.3)	0.710
Dyspnea, n(%)	680 (42.8)	396 (43)	284 (42.5)	0.828
No symptoms, n(%)	587 (36.9)	349 (37.9)	238 (35.6)	0.345
*Medical therapy*				
ACEi/ARBs, n(%)	1066 (67)	608 (66)	458 (68.5)	0.306
Beta-blockers, n(%)	971 (61.1)	557 (60.5)	414 (61.9)	0.571
Statins, n(%)	974 (61.2)	557 (60.5)	417 ((62.3)	0.454
Antiplatelet, n(%)	1027 (64.6)	577 (62.6)	450 (67.3)	0.058
Oral anticoagulant, n(%)	326 (20.5)	180 (19.5)	146 (21.8)	0.267
*Laboratory tests*				
Creatinine, mg/dL	0.92 (0.76–1.1)	0.92 (0.75–1.1)	0.92 (0.77–1.1)	0.365
Hemoglobin, g/dl	13.5 (12–14.5)	13.5 (12–14.6)	12.5 (12–14)	0.891
Platelets, × 1000	225 (186–275)	228 (188–270)	219 (180–286)	0.722
AST, IU/L	19 (16–23)	19 (16–24)	19 (15–23)	0.346
ALP, IU/L	17 (14–22)	17 (14–22)	16 (14–22)	0.053
*Echocardiographic parameters*				
LVEDV, ml	96 (85–117)	96 (85–118)	96 (86–117)	0.857
LVEF, %	55 (51–55)	55 (51–55)	52 (47–53)	0.328
Diastolic dysfunction grade II, n(%)	369 (23.2)	196 (21.3)	173 (25.9)	0.035
Diastolic dysfunction grade III, n(%)	34 (2.1)	27 (2.9)	7 (1.5)	0.013
TAPSE, mm	24 (21–27)	24 (21–27)	22 (20–26)	0.298
SPAP, mmHg	30 (25–30)	30 (25–30)	28 (24–28)	0.130
*Outcomes*				
Composite endpoint, n(%)	39 (2.4)	31 (3.4)	8 (1.2)	0.006
Acute coronary syndrome, n(%)	31 (1.9)	24 (2.6)	7 (1.04)	0.027
Myocardial infarction, n(%)	20 (1.3)	16 (1.4)	4 (0.6)	0.065
STEMI, n(%)	3 (0.2)	3 (0.2)	0 (0)	n.a
NSTEMI, n(%)	17 (1.1)	14 (1.2)	4 (0.6)	0.225
Unstable angina, n(%)	11 (0.6)	8 (0.8)	3 (0.5)	0.7
Cardiac death, n(%)	3 (0.3)	3 (0.3)	0 (0)	n.a
Cardiogenic pulmonary edema, n(%)	3 (0.3)	2 (0.2)	1 (0.1)	0.643
Cardiogenic shock, n(%)	2 (0.2)	2 (0.2)	0 (0)	n.a

Bold indicates that the *p* value reached statistical significance with *p* value < 0.05

*ACEi* angiotensin-converting enzyme inhibitors, *ALP* alkaline phosphatase, *ARBs* angiotensin receptor blockers, *AST* aspartate aminotransferase, *ASA* American Society of Anesthesiologists, *CABG* coronary artery bypass graft, *CAD* coronary artery disease, *CKD* chronic kidney disease, *LVEDV* left ventricular end-diastolic volume, *LVEF* left ventricular ejection fraction, *METS* metabolic equivalents, *MPI* myocardial perfusion imaging, *NSTEMI* non-ST-segment elevation myocardial infarction, *NYHA* New York Heart Association, *PCI* percutaneous coronary intervention, *SPAP* systolic pulmonary artery pressure, *STEMI* ST-segment elevation myocardial infarction, *TAPSE* tricuspid annular plane systolic excursion

The median age of the patients was 71 years (65–77), and 38.8% (617) were female. Regarding cardiovascular risk factors, 81.1% (1290) of the patients had hypertension, 69.2% (1100) dyslipidemia, and 32.7% (520) diabetes mellitus. Moreover, 43.7% (695) of patients had a history of CAD; moderate-to-severe CKD had a prevalence of 16.3% (260).

Concerning symptoms, 42.8% (680) of patients suffered from dyspnea, 4.2% (67) from typical angina, and 26.8% (426) from atypical chest pain.

Electrocardiogram and echocardiogram were available in all patients: 94% (1495) were in sinus rhythm and 6% (95) in atrial fibrillation. Median left ventricular ejection fraction (LVEF) was 55% (51–55). There was a slightly greater incidence of grade II diastolic dysfunction in the stress-tested group (25.9% vs. 21.3%,* p* 0.035), whereas grade III dysfunction was more prevalent in the non-stress group (2.9% vs. 1.5%, *p* 0.013).

### *Type of surgery and surgical risk scores *( Fig. 2)

**Fig. 2:**
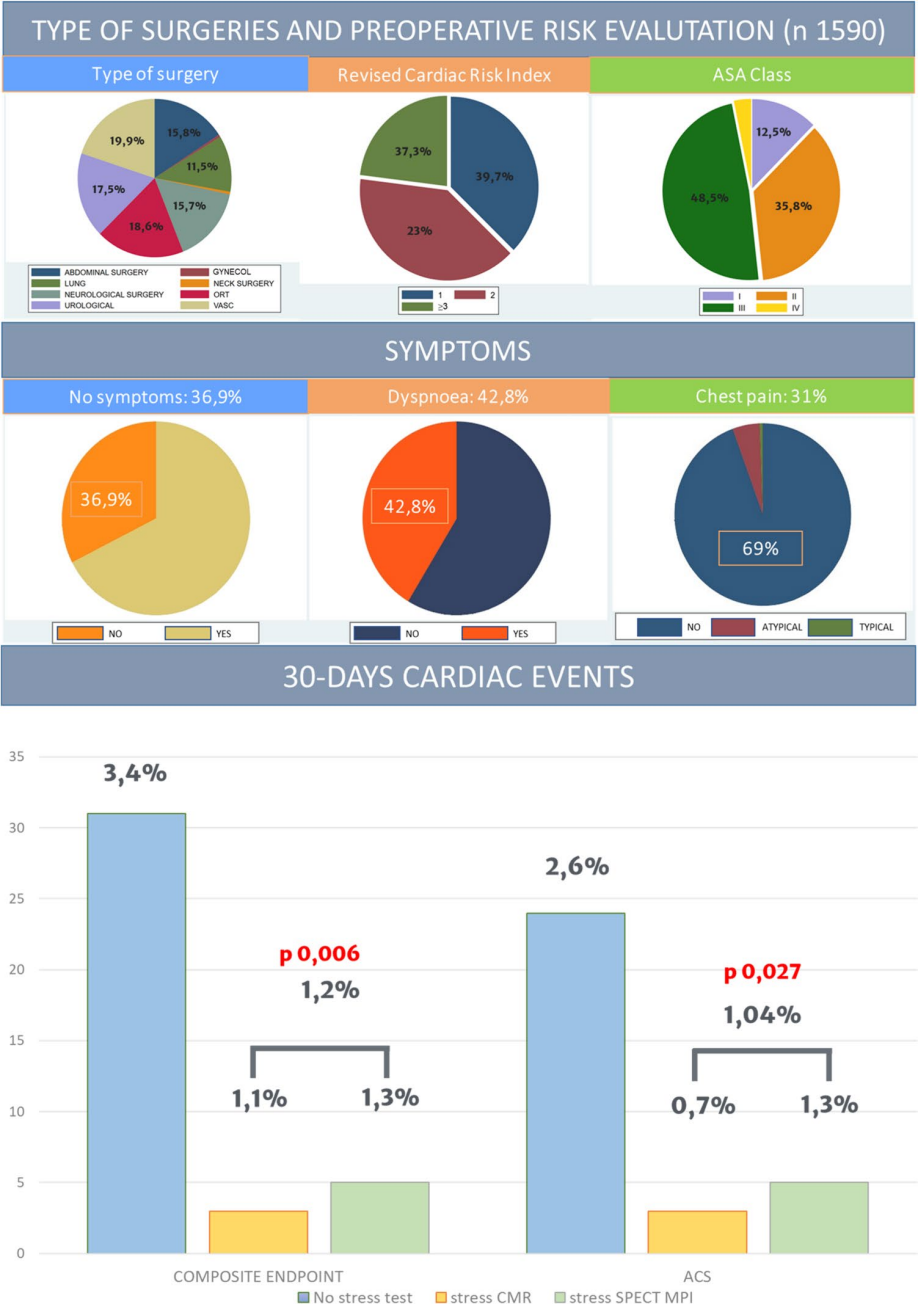
Type of surgeries, preoperative risk evaluation, and 30-day cardiac events. The most prevalent type of non-cardiac surgery was vascular (19.9%), followed by orthopedic (18.6%). About the half of surgeries were at high risk. Revised cardiac index was high (≥ 3) in 37.3% of patients, and ASA class III was present in 48.5% of cases. The study population was divided in two groups (no stress test vs. imaging stress test). Primary composite endpoint and secondary endpoint (ACS) were significantly lower in the imaging stress-tested group. *ACS* acute coronary syndrome, *ASA* American Society of Anesthesiologists, *CMR* cardiac magnetic resonance, *GYNECOL* gynecological surgery, *ORT* orthopedic surgery, *SPECT-MPI* single-photon emission computed tomography myocardial perfusion imaging, *VASC* vascular surgery

The most prevalent type of NCS was vascular (n = 316, 19.9%), followed by orthopedic (n = 296, 18.6%) and urological (n = 278, 17.5%). Almost half of surgeries were at high risk (50,1%). RCRI was calculated for each patient: 37.4% (595) had an index of 1, 39.6% (630) of 2, and 23% (366) had an index of more than 3. With regard to ASA class, 12.5% (199) of patients were in class I; 35.7% (567) in class II; 48.5% (772) in class III, and 3.2% (51) in ASA class IV.

### Non-invasive imaging cardiac test

A total of 669 patients underwent a stress test before NCS. Results and outcomes of sCMR and SPECT-MPI are shown in Table 2; baseline clinical characteristics are available in supplemental Table 1. A sCMR was performed in 287 patients. Mean LVEF was 58.7% (± 10), and late gadolinium enhancement was present in 39.7% of cases. Inducible ischemia was detected in 52/287 patients (18.1%). The median value of ischemic segments was 3 (2–4), with a total percentage of stress myocardial ischemia of 8.6% (± 3.4).

**Table 2:** MPI stress test results and outcomes for sCMR and SPECT-MPI. Values are indicated as number (n) and percentage (%), mean ± SD or median and interquartile range

	Stress CMR (287 patients)	Stress SPECT-MPI (382 patients)	*p* value
*MPI test results*			
Positive result, n(%)	52 (18.1)	49 (12.8)	0.059
Percentage of stress ischemia, %	8.6 ± 3.4	8.2 ± 4.4	0.515
Number of stress ischemic segments	3 (2–4)	4 (2–4)	0.429
Mild ischemia, n (%)	9 (3.1)	10 (2.6)	0.68
Moderate–severe ischemia, n (%)	43 (14.9)	39 (10.2)	0.06
Presence of LGE and Ischemia, n (%)	34 (11.8)	n.a	n.a
Presence of LGE at CMR, n(%)	114 (39.7)	n.a	n.a
–ischemic LGE	76 (67)		
–non-ischemic LGE	38 (33)		
Number of CMR LGE segments	2 (1–3)	n.a	n.a
Presence of rest and stress ischemia at SPECT, n (%)	n.a	30 (7.9)	n.a
Presence of rest ischemia at SPECT, n(%)	n.a	119 (31.2)	n.a
Percentage of SPECT rest ischemia, %	n.a	6.1 ± 3.1	n.a
Number of SPECT rest ischemic segments	n.a	3 (2 – 5)	n.a
LVEF, %	58.7 ± 10.0	61.9 ± 9.7	**0.001**
LVEDV, ml	141 ± 37.8	135 ± 26.8	0.061
ICA, n(%)	54 (18.8)	51 (13.3)	0.054
Revascularized patients, n(%)	44 (15.3)	38 (9.7)	0.036
Rate of revascularization in patients with reversible ischemia, n(%)	44 (84.5)	38 (77.6)	0.448
*Outcomes*			
Composite endpoint, n(%)	3 (1.1)	5 (1.3)	0.756
Acute coronary syndrome, n(%)	2 (0.7)	5 (1.3)	0.441
Myocardial infarction, n(%)	1 (0.3)	3 (0.8)	0.638
STEMI, n(%)	0 (0.0)	0 (0.0)	n.a
NSTEMI, n(%)	1 (0.3)	3 (0.8)	0.638
Unstable angina, n(%)	1 (0.3)	2 (0.5)	1.000
Cardiogenic pulmonary edema, n(%)	1 (0.3)	0 (0.0)	0.429

Bold indicates that the p value reached statistical significance with *p* value < 0.05

*ICA* invasive coronary angiography, *LGE* late gadolinium enhancement, *LVEDV* left ventricular end-diastolic volume, *LVEF* left ventricular ejection fraction, *MPI* myocardial perfusion imaging; n.a. not applicable, *NSTEMI* non-ST-segment elevation myocardial infarction, *sCMR* stress cardiac magnetic resonance, *SPECT* single-photon emission computed tomography, *STEMI* ST-segment elevation myocardial infarction

A SPECT-MPI was performed in 382 patients and resulted positive for inducible ischemia in 49/382 cases (12.8%). The median value of reversible stress ischemic segments was 4 (2–4) with a total percentage of stress myocardial ischemia of 8.2% (± 4.4). Rest ischemia was detected in 31.2% of patients. Physical stress was performed in 57% of cases, while pharmacological in 43% of cases.

Indication to ICA after stress imaging was upon clinical judgment of the referring cardiologist.

### Invasive coronary angiography

In the stress-tested group, a total of 105 patients (15.7%) underwent ICA before NCS and 82 (78% of the patients who underwent ICA) were revascularized. The rate of ICAs was not significantly different between sCMR and SPECT-MPI groups (18.8% vs. 13.3%, respectively, 0.054), despite a tendency toward higher rate of ICA in sCMR group with a similar rate of revascularizations (84.5% vs. 77.6%, respectively, *p* 0.448).

### Myocardial ischemia predictors in patients undergoing non-cardiac surgery

Univariate and multivariate logistic regression analysis was performed to assess the association between clinical and echocardiographic parameters and the presence of myocardial ischemia in patients undergoing MPI stress test (Table 3 and supplemental Table 2). Independent predictors of myocardial ischemia were hypertension (OR 5.72, 95%CI 1.70–19.15, *p* 0.005), male sex (OR 1.74, 95% CI 1.09–2.79, *p* 0.020), moderate–severe CKD (OR 1.74, 95%CI 1.06–2.84, *p* 0.026), known CAD (OR 4.01, 95%CI 2.30–6.99, *p* < 0.001) and high-grade diastolic dysfunction (OR 1.85, 95%CI 1.30–2.64, *p* 0.001).

**Table 3:** Independent variables associated with the composite primary endpoint (3.A) and acute coronary syndromes (3.B). Predictors of ischemia at MPI stress test (3.C)

	OR (95% CI)	*p* value
*3.A Multivariable analysis for primary composite endpoint*		
Coronary artery disease	2.33 (1.11–4.88)	**0.025**
Moderate–severe CKD	1.79 (0.85–3.78)	0.122
NYHA class	1.03 (0.58–1.82)	0.917
Revised cardiac risk index score	1.07 (0.67–1.69)	0.769
Predicted METS	0.26 (0.12–0.55)	**0.001**
Stress test strategy	0.33 (0.15–0.76)	**0.009**
*3.B Multivariable analysis for ACS*		
Coronary artery disease	2.77 (1.18–6.48)	**0.019**
Moderate–severe CKD	1.29 (0.53–3.14)	0.572
NYHA class	1.12 (0.59–2.12)	0.706
Revised cardiac risk index score	1.02 (0.61–1.71)	0.926
Predicted METS	0.20 (0.09–0.47)	**< 0.001**
Stress test strategy	0.41 (0.17–0.98)	**0.046**
*3.C Multivariable analysis for Ischemia at MPI stress tests*		
Male	1.74 (1.09–2.79)	**0.020**
Dyslipidemia	0.55 (0.30–1.00)	0.051
Diabetes	1.04 (0.66–1.62)	0.863
Hypertension	5.72 (1.70–19.15)	**0.005**
Coronary artery disease	4.01 (2.30–6.99)	**< 0.001**
Moderate–severe CKD	1.74 (1.06–2.84)	**0.026**
Revised cardiac risk index score	1.24 (0.91–1.70)	0.162
Diastolic dysfunction II,III	1.85 (1.30–2.64)	**0.001**

Bold indicates that the *p* value reached statistical significance with *p* value < 0.05

*ACS* acute coronary syndromes, *CKD* chronic kidney disease, *CI* confidence intervals, *METS* metabolic equivalents, *MPI* myocardial perfusion imaging, *NYHA* New York Heart Association, *OR* odds ratio

### Myocardial ischemia and revascularization

**Fig. 3:**
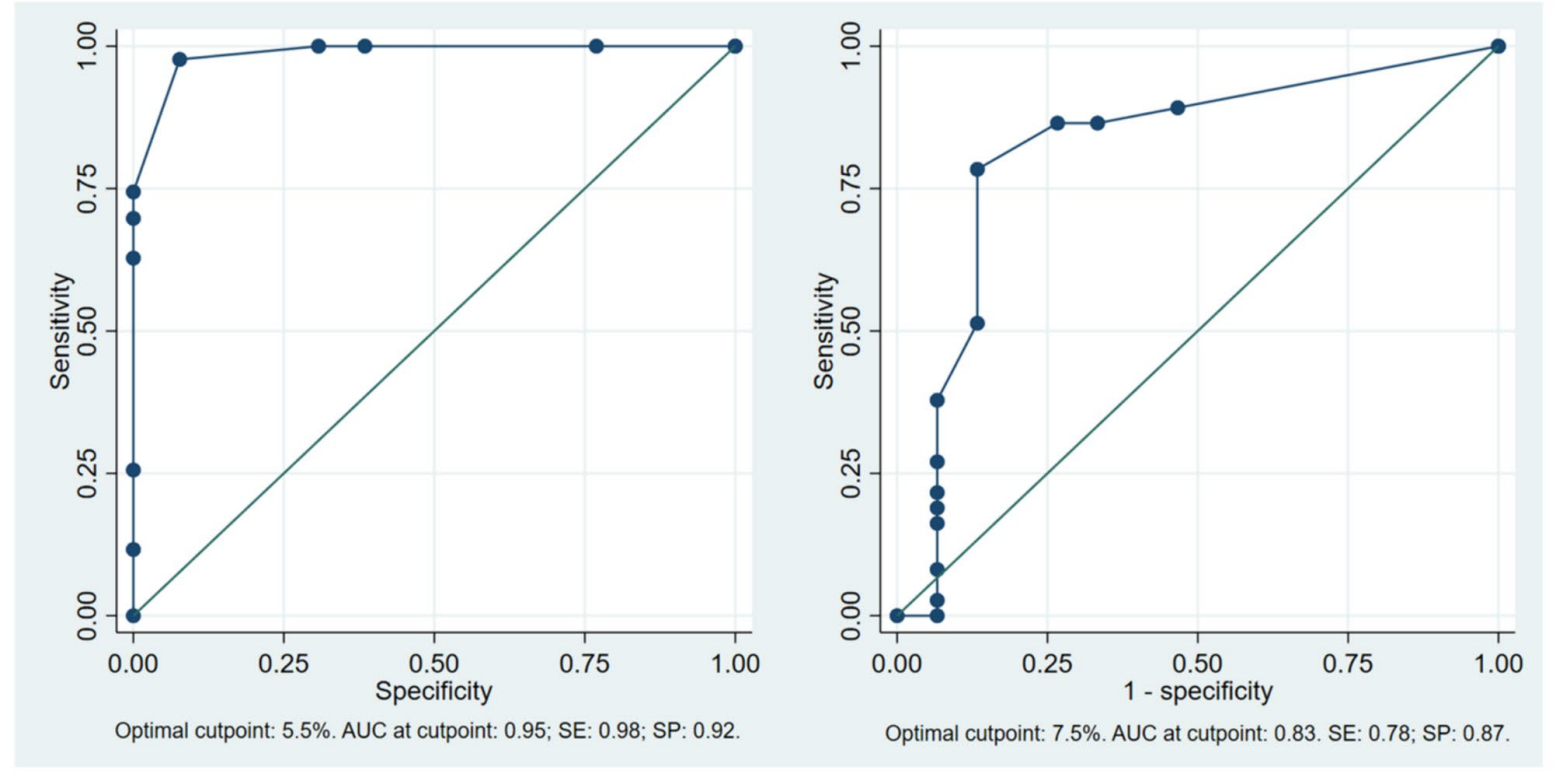
ROC, AUC, and the optimal cut-points of sCMR and SPECT-MPI to predict coronary artery revascularization. *AUC* area under the curve, *ROC* receiver operating characteristics, *sCMR* stress cardiac magnetic resonance, *SE* sensitivity, *SP* specificity, *SPECT-MPI* single-photon emission computed tomography myocardial perfusion imaging

AUC and the optimal cut-points were calculated both for sCMR and SPECT-MPI to identify the best ischemic thresholds for revascularization (Fig. 3). Stress CMR showed an AUC of 0.95 (SE: 0.98, SP: 0.92) when a threshold for myocardial ischemia of 5.5% was chosen. On the other side, the best cut-point for SPECT-MPI was a percentage of ischemia of 7.5% (AUC: 0.83, SE: 0.78, SP: 0.87).

### Cardiac events in non-cardiac surgery

The composite primary outcome occurred in 39 cases (2.4%) with a significantly greater incidence in the non-stress-tested group compared to the stress-tested group (respectively, 3.4% vs. 1.,2%, *p* 0.006; Table 1 and Fig. 2). Myocardial infarction was the most frequent cardiac complication (n = 20, 1.3% of cases), followed by unstable angina (n = 11, 0.6%), cardiac death (n = 3, 0.3%), cardiogenic pulmonary edema (n = 3, 0.3%), and cardiogenic shock (n = 2; 0.2%). ACS occurred more frequently in the non-stress-tested group (2.6% vs. 1.0%; *p* 0.027; Table 1 and Fig. 2).

Both primary outcome and ACS did not differ between sCMR and SPECT (primary outcome: 1.1% vs. 1.3%, *p* 0.756; ACS 0.7% vs. 1.3% *p* 0.441; Table 2).

**Fig. 4:**
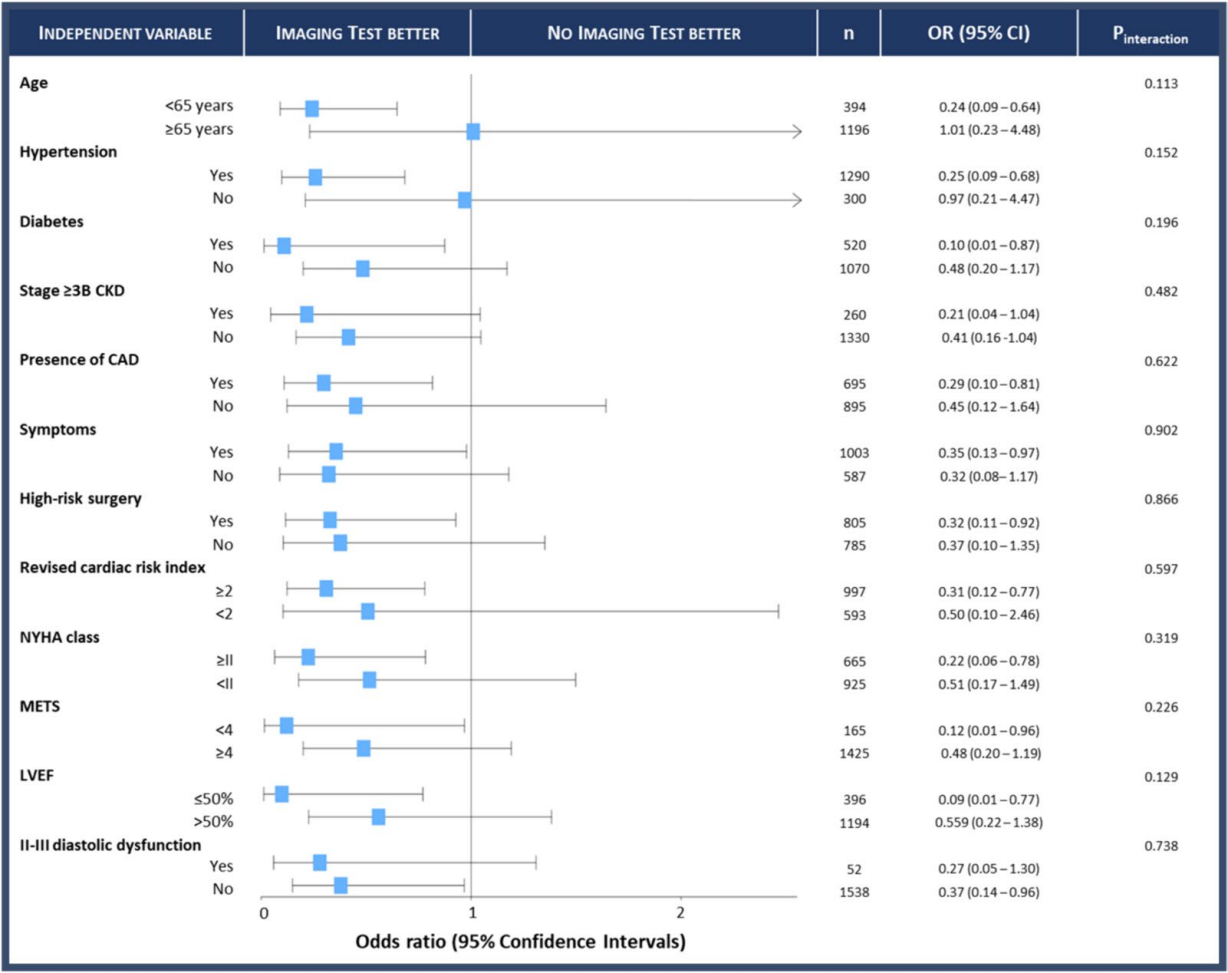
Test for interaction of imaging stress test strategy among different subgroups. The independent protective effect of the MPI stress test strategy was consistent among all sub-categories. *CAD* Coronary artery disease, *CKD* chronic kidney disease, *CI* confidence intervals, *LVEF* left ventricular ejection fraction, *METS* metabolic equivalents, *NYHA* New York Heart Association, *OR* odds ratio

After adjusting with multivariable logistic regression analysis, CAD (OR 2.33, 95%CI 1.11–4.88, *p* 0.025) was associated with an increased risk of the primary study endpoint, while higher METs grade (OR 0.26, 95%CI 0.12–0.55, *p* 0.001) and a stress test strategy (OR 0.33, 95%CI 0.15–0.76, *p* 0.009) were protective (Table 3 and supplemental Table 3). The independent protective effect of the MPI stress test strategy was consistent among all sub-categories, as shown by test for interaction (Fig. 4). Similarly, we performed a multivariable logistic regression analysis for the secondary endpoint ACS (Table 3). The protective effect of stress test strategy was confirmed also for ACS alone (OR 0.41, 95%CI 0.17–0.98, *p* 0.046).

### Propensity score analysis

Propensity score matching was utilized to create a balanced comparison, resulting in a cohort of 669 matched pairs. After matching, the baseline characteristics between the stress-tested and non-tested groups showed no significant differences, with the largest standardized mean difference being 0.08 for the presence of diabetes. The lower rate of postoperative cardiovascular events in the matched stress-tested group suggests a potential benefit of preoperative stress testing in reducing these events. The C-index values for different models were as follows: non-MPI (0.65), sCMR with revascularization (0.85), sCMR without revascularization (0.80), SPECT with revascularization (0.82), SPECT without revascularization (0.75). ROC analysis demonstrated AUC values ranging from 0.66 for non-MPI to 0.86 for sCMR with revascularization. The DeLong test indicated that sCMR with revascularization had significantly higher AUC compared to SPECT with revascularization (*p* = 0.03). Post-matching analysis indicated that the rate of 30-day postoperative cardiovascular events in the stress-tested group was significantly lower than the non-tested group (OR 0.34; 95% CI, 0.15–0.78; *p* = 0.009) and similarly resulted for the incidence of myocardial infarction (*p* = 0.046). When examining the outcomes of patients with positive stress test results, 18.1% in the sCMR group and 12.8% in the SPECT-MPI group had inducible ischemia, leading to ICA in 78% and a subsequent revascularization rate of 84.5% for the sCMR group and 77.6% for the SPECT-MPI group. The matched cohort analysis mitigates the effects of referral bias and provided a more accurate estimate of the impact of preoperative stress testing, overcoming the selection bias intrinsic to the retrospective design of the study. The lower rate of postoperative cardiovascular events in the matched stress-tested group also confirms a potential benefit of preoperative stress testing in reducing these events.

## Discussion

**Fig. 5:**
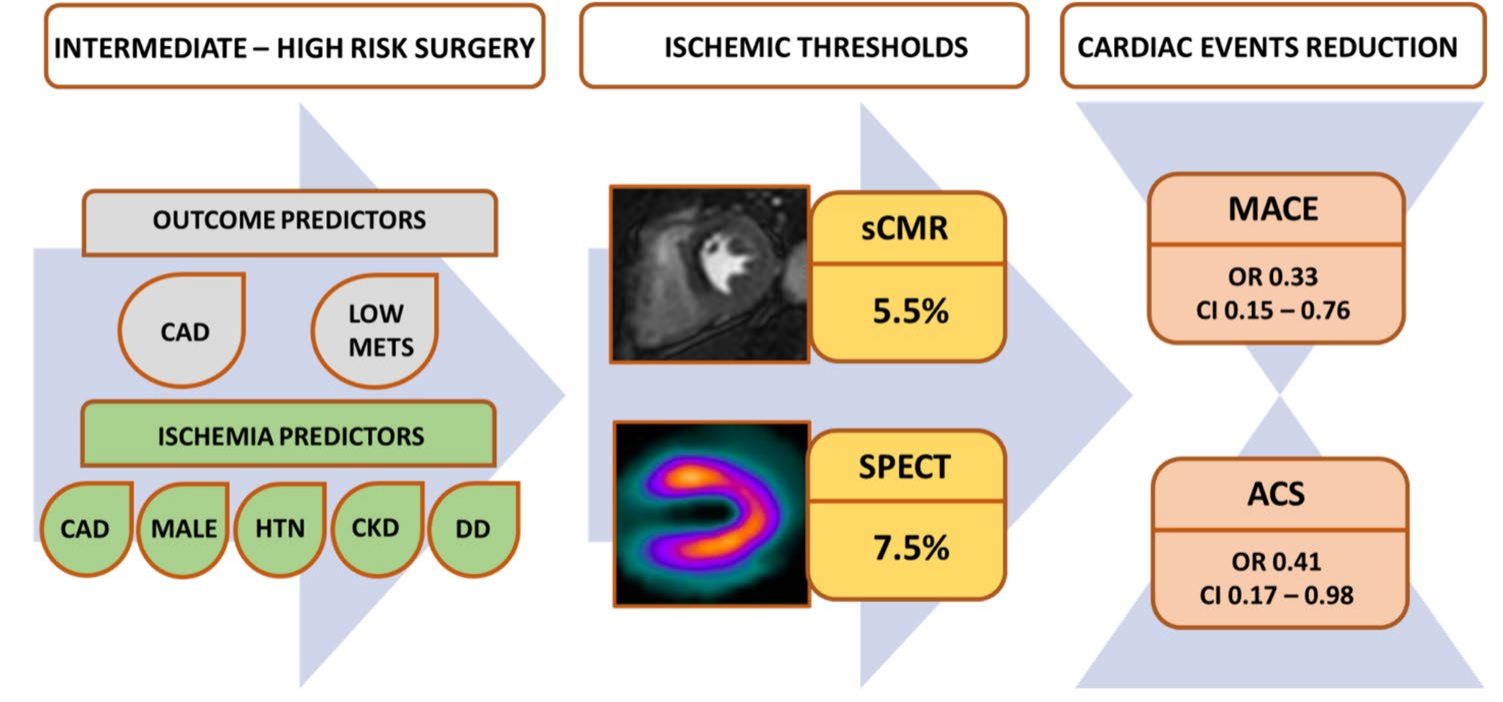
Proposed algorithm to select patients who may benefit from a pre-operative stress test strategy. Both outcomes predictors and ischemic predictors must be considered during the preoperative evaluation before NCS. The suggested ischemic thresholds are 5.5% for sCMR and 7.5% for SPECT. The imaging stress test strategy is associated with a reduction in both MACE and ACS in the 30-day postoperative period. *ACS* acute coronary syndrome, *CAD* coronary artery disease, *CI* confidence intervals, *CKD* chronic kidney disease, *DD* diastolic dysfunction grade II and III, *HTN* hypertension, *MACE* major adverse cardiac events, *METS* metabolic equivalents, *NCS* non-cardiac surgery, *OR* odds ratio, *sCMR* stress cardiac magnetic resonance, *SPECT* single-photon emission computed tomography

**Fig. 6:**
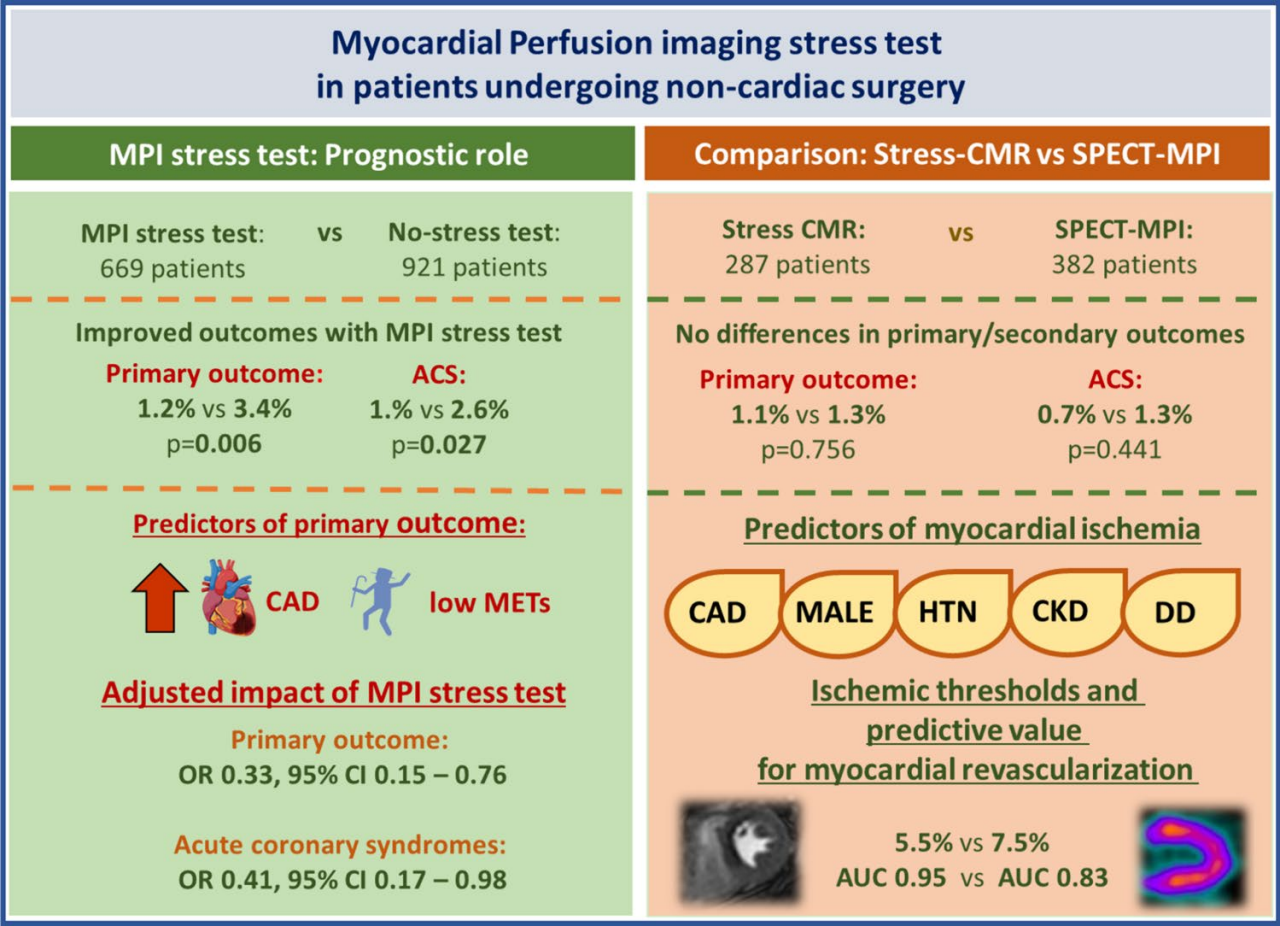
Summary of study results. ACS: acute coronary syndrome. AUC: area under the curve. CAD: coronary artery disease. CI: confidence intervals. CMR: stress cardiac magnetic resonance. CKD: chronic kidney disease. DD: diastolic dysfunction grade II and III. HTN: hypertension. METS: metabolic equivalents. MPI: myocardial perfusion imaging. NCS: non-cardiac surgery. OR: odds ratio. SPECT: single-photon emission computed tomography

The aim of the present study was to assess the perioperative risk stratification value of a stress imaging test strategy with sCMR or SPECT-MPI in patients with ≥ 2 risk factors or known CAD undergoing intermediate-to-high-risk NCS. The main findings may be summarized as follows (Figs. 5 and 6):

- A MPI stress test strategy, irrespective of the imaging modality used, was associated with a significantly reduced risk of perioperative cardiac complications;
- Known CAD is an independent predictor of perioperative cardiac complications, while predicted METs and a MPI stress test strategy are independent protective factors;
- The incidence of perioperative cardiac complications was comparable between patients undergoing sCMR and SPECT-MPI;
- In the setting of perioperative risk stratification before NCS, the discriminative power of MPI ischemic threshold for coronary revascularization is excellent for sCMR (cut-point 5.5%) and good for SPECT-MPI (cut-point 7.5%).

To further enhance the discriminative capability of different models with respect to the outcomes, we performed C-index analysis and compared the AUC values of different models using the DeLong test to evaluate whether the differences in discriminative ability were systematic or random. Results of ROC curves and AUC values demonstrated statistical differences in the discriminative capabilities of the models. The DeLong test confirmed that the variations in AUC values among the models were significant, indicating systematic differences in their discriminative abilities. This additional analysis underscores the discriminative power of sCMR and SPECT-MPI stress tests in predicting perioperative cardiac complications, thereby supporting their potential use in clinical practice for patients undergoing intermediate-to-high-risk NCS. Recently published guidelines [1] suggest the use of stress imaging test before high-risk elective NCS in patients with poor functional capacity and high likelihood of CAD or high cardiovascular risk, even if asymptomatic, to intercept patients with unfavorable trajectory after NCS. However, patients’ selection for stress imaging before NCS is often challenging in clinical practice, as the functional capacity and symptomatic state of surgical patients are difficult to evaluate due to the underlying diseases (i.e., cancer, orthopedic injuries, limited mobility). Moreover, current guidelines do not provide indications for specific imaging modalities, and existing evidence primarily focuses on stress echocardiography or SPECT-MPI [13–18]. Exercise stress test without imaging did not show promising results before non-cardiac surgery and guidelines do not recommend its use if an imaging test is available [1]. Resting transthoracic echocardiography is currently recommended to identify patients with severe valvular disease or cardiomyopathy, but its role for risk prediction before NCS is limited [19, 20]. Left ventricular ejection fraction is considered a borderline predictor of cardiac events, whereas diastolic dysfunction was identified as a significant predictor of events in several studies and one large meta-analysis [21]. In our study, all patients underwent an echocardiographic evaluation before surgery and, while none of the echocardiographic parameters were associated with the clinical endpoints, diastolic dysfunction (grade II and III) resulted as an independent predictor of myocardial ischemia, together with hypertension, known CAD, male sex and chronic kidney disease. Thus, our findings suggest considering stress imaging in patients with high-grade diastolic dysfunction to optimize risk stratification before surgery.

Most of published studies are focused on stress echocardiography and SPECT-MPI [13–18, 22]; there is general agreement in considering the absence of inducible ischemia as a marker of favorable clinical outcomes after NCS [23, 24]. However, a recent meta-analysis has shown that currently available evidence is not sufficient to demonstrate a benefit of a stress test strategy before NCS. Notably, only 5 of the 76 included studies in this meta-analysis compared imaging stress test vs. non-stress-tested groups, and none used sCMR [2]. In our study, adopting a stress imaging strategy with sCMR or MPI-SPECT, which targeted coronary revascularization, was independently associated with a significant risk reduction in cardiac events (OR 0.33; 95%CI 0.15–0.76). Of note, our study represents the first evidence for the use of sCMR in this scenario and demonstrates that sCMR has a greater accuracy than SPECT in terms of discriminative power of ischemic threshold for revascularization, together with known advantages of lower acquisition times and no radiation exposure related to the modality.

According to a meta-analysis including 79 studies and 1179 stable patients undergoing SPECT-MPI, less than 20% inducible myocardial ischemia does not significantly portend an increased risk of cardiac complications[25]. However, the impact of revascularization in this setting was not investigated and an ischemia threshold to treat patients undergoing NCS can only be hypothesized. In the general population, more than 10% of myocardial ischemia on nuclear imaging and at least 2 ischemic segments on sCMR are considered the reference thresholds for revascularization [26]. In our real-life retrospective study, referring cardiologists did not use a fixed cutoff value to refer patients to ICA, so some patients were scheduled for ICA with evidence of mild or moderate ischemia. The mean ischemic myocardial percentages were 8.6% and 8.2% for sCMR and SPECT, respectively, while the best accuracy thresholds were 5.5% for sCMR (AUC 0.95) and 7.5% for SPECT (AUC 0.83). Even if these cutoff values need a prognostic validation, they could be used to select patients who may benefit most from a pre-operative ICA, preventing useless delay of surgical intervention in case of false positive results. Our results may suggest a revision of current guidelines regarding revascularization treatment prior to NCS. A personalized approach can be favorable for these patients’ outcome, as the stress of surgery can exacerbate underlying cardiovascular conditions, requiring tailored ischemia thresholds and stress management. Currently, there is paucity of information regarding the role of ICA before NCS and the same recommendation of the non-surgical setting are used [1]. While there are many observational or non-randomized studies that suggest to treat moderate and severe ischemia to improve symptoms and quality of life in patients not presenting with ACS, recent results of large randomized trials (COURAGE and ISCHEMIA) showed no benefit of an early revascularization strategy in stable patients based on ischemia assessment in terms of myocardial infarction, cardiac death, and hospitalizations [27–29]. Moreover, in the setting of preoperative cardiological assessment before elective vascular surgery, CARP trial demonstrated no benefit of coronary revascularization in short- and long-term myocardial infarction mortality [30].

However, the pre-operative evaluation before NCS is a different scenario due to the planned surgical procedure itself, which exposes patients’ cardiovascular system to oxygen supply–demand imbalance, hemodynamic changes, as well as prothrombotic and pro-inflammatory states [23]. As reported in one large registry, major NCS is associated with a perioperative major adverse cardiovascular events incidence of about 3% [31]. A large prospective multicenter study showed an even higher incidence (13.1%) of myocardial infarction/injury with an extended 1-year follow-up [32]. In our study, including only intermediate-to-high-risk surgery, the overall incidence of 30-day cardiac events was 2.4%, and it dropped down to 1.2% in the MPI stress test strategy group. In conclusion, although the clinical impact of inducible ischemia in patients with known or suspected stable CAD has been largely questioned by recent trials, our results support the role of a MPI stress test, particularly sCMR, in the specific setting of pre-operative risk stratification for intermediate-to-high-risk NCS. Accordingly, we propose an algorithm to select patients who may benefit from a pre-operative stress test strategy (Fig. 5): along with outcomes predictors (CAD, low METS), ischemia predictors must be considered to further improve risk stratification before NCS (male sex, hypertension, CKD and diastolic dysfunction grade II and III). Stress CMR should be preferred, when available, over SPECT-MPI and an ischemia threshold of 5.5% is recommended as gatekeeper to ICA.

## Study limitations

Our study should be interpreted considering some limitations. First, its single-center and observational retrospective nature determines intrinsic limitations in terms of generalizability and potential selection bias. However, we analyzed a wide population of moderate-to-high-risk patients candidates to different types of intermediate-to-high-risk surgery in a high-volume tertiary center. Moreover, the single-center fashion increased the reproducibility in terms of indications and results of stress imaging among different patients and propensity score analysis was performed to address potential referral/selection bias. Secondly, serum cardiac biomarkers were not systematically assessed and are not reported. The recommendation of a systematic assessment of troponin and BNP in patients undergoing NCS has been recently provided by ESC Guidelines, while our study focuses on a previous period. Third, the relatively small number of events in the MPI stress-tested group prevented us from further exploring any difference between sCMR and SPECT-MPI in terms of clinical events and might have led to possible type II error for the primary outcome. Notwithstanding, our study represents one of the largest single-center analyses exploring the role of an MPI stress test strategy in patients undergoing NCS and is the first study reporting data on the sCMR use in this setting. A final limitation regards the methodological discrepancies in terms of stressors (regadenoson vs. adenosine vs. physical effort). Nevertheless, our approach aligns with clinical guidelines and reflects a real-world clinical approach.

## Conclusions

A stress imaging strategy is associated with a reduced incidence of cardiac events in high-risk patients candidate to intermediate-to-high-risk non-cardiac surgery. The probability of cardiac complications is similarly reduced by sCMR and SPECT-MPI, although sCMR is more accurate in predicting coronary artery revascularizations. Future randomized clinical trials are required to confirm and validate our findings.

## Supplementary Information

Below is the link to the electronic supplementary material.

Supplementary file1 (DOCX 32 KB)

## Abbreviations

NCS: Non-cardiac surgery
ACEi/ARBs: Angiotensin-converting enzyme inhibitors/angiotensin II receptor blockers
ACS: Acute coronary syndromes
ALP: Alkaline phosphatase
ASA: American Society of Anesthesiologists
AST: Aspartate aminotransferase
AUC: Area under the curve
BNP: B-type natriuretic peptide
CABG: Coronary artery bypass grafting
CAD: Coronary artery disease
CCTA: Coronary computed tomography angiography
CKD: Chronic kidney disease
ECG: Electrocardiogram
ESC: European Society of Cardiology
ICA: Invasive coronary angiography
IQR: Interquartile range
LGE: Late gadolinium enhancement
LVEDV: Left ventricular end-diastolic volume
LVEF: Left ventricular ejection fraction
METS: Metabolic equivalents
MPI: Myocardial perfusion imaging
NYHA: New York Heart Association
PCI: Percutaneous coronary intervention
RCRI: Revised cardiac risk index
ROC: Receiver operating characteristic
sCMR: Stress cardiac magnetic resonance
SD: Standard deviation
SPAP: Systolic pulmonary arterial pressure
SPECT-MPI: Single-photon emission computed tomography myocardial perfusion imaging
TAPSE: Tricuspid annular plane systolic excursion
