# Supplementary material for: Prognostic value of stress CMR and SPECT-MPI in patients undergoing intermediate-to-high-risk non-cardiac surgery
Source: Radiol Med. 2024 Sep 10;129(10):1485–98. doi: 10.1007/s11547-024-01876-x (PMC11480140; doi:10.1007/s11547-024-01876-x)
Supplement: Supplementary file 1 — Supplementary file1 (DOCX 32 KB) [file 11547_2024_1876_MOESM1_ESM.docx]

**TITLE:** Prognostic value of stress-CMR and SPECT-MPI in patients undergoing intermediate-to-high risk non-cardiac surgery.

**Supplemental Table 1.** Clinical characteristics of the MPI-stress test group divided in stress CMR vs stress SPECT-MPI. Values are indicated as number (n) and percentage (%), mean ± SD or median (interquartile range). Bold indicates that the p value reached statistical significance with p value < 0.05. ACEi: Angiotensin-converting enzyme inhibitors. ALP: alkaline phosphatase. ARBs: Angiotensin receptor blockers. AST: aspartate aminotransferase. ASA: American Society of Anesthesiologists. CABG: coronary artery bypass graft. CAD: coronary artery disease. CKD: chronic kidney disease. DCM: dilated cardiomyopathy. ICD: implantable cardioverter defibrillator. LVEDV: left ventricular end diastolic volume. LVEF: left ventricular ejection fraction. METS: metabolic equivalents. MPI: myocardial perfusion imaging. n.a.: not applicable. NYHA: New York Heart Association. PCI: percutaneous coronary intervention. sCMR: stress cardiac magnetic resonance. SPAP: systolic pulmonary artery pressure. SPECT: single photon emission computed tomography. TAPSE: tricuspid annular plane systolic excursion.

**Supplemental Table 2.** Predictors of ischemia at MPI-stress test. Univariable and multivariable analysis. Bold indicates that the p value reached statistical significance with p value < 0.05. ACEi: Angiotensin-converting enzyme inhibitors. ARBs: Angiotensin receptor blockers. ASA: American Society of Anesthesiologists. CABG: coronary artery bypass graft. CAD: coronary artery disease. CKD: chronic kidney disease . CI: confidence intervals. DCM: dilated cardiomyopathy. LVEF: left ventricular ejection fraction. METS: metabolic equivalents. MPI: myocardial perfusion imaging. n.s.: not significant. NYHA: New York Heart Association. OR: odds ratio. PCI: percutaneous coronary intervention.

**Supplemental Table 3.** Independent variables associated to the composite primary endpoint. Univariable and multivariable analysis. Bold indicates that the p value reached statistical significance with p value < 0.05. ACEi: Angiotensin-converting enzyme inhibitors. ARBs: Angiotensin receptor blockers. ASA: American Society of Anesthesiologists. CABG: coronary artery bypass graft. CAD: coronary artery disease. CKD: chronic kidney disease. CI: confidence intervals. DCM: dilated cardiomyopathy. LVEF: left ventricular ejection fraction. METS: metabolic equivalents. MPI: myocardial perfusion imaging. n.s.: not significant. NYHA: New York Heart Association. OR: odds ratio. PCI: percutaneous coronary intervention.

| **Supplemental table 1** | **Stress CMR**  **(287 patients)** | **Stress SPECT-MPI**  **(382 patients)** | **p-value** |
| --- | --- | --- | --- |
| **Demographic and clinical characteristics** | | | |
| Age | 68 (59-63) | 74 (67-78) | 0.064 |
| Female, n (%) | 106 (36.9) | 147 (38.5) | 0.491 |
| Familiar History of CAD, n (%) | 48 (16.7) | 75 (19.6) | 0.883 |
| Hypertension, n (%) | 201 (70.0) | 334 (87.4) | 0.313 |
| Dyslipidemia, n (%) | 166 (57.8) | 293 (76.7) | 0.674 |
| Diabetes, n (%) | 74 (25.8) | 142 (37.2) | 0.762 |
| Current or former Smoking, n (%) | 164 (57.0) | 241 (63.0) | 0.896 |
| Coronary artery disease, n (%) | 119 (41.5) | 191 (50.0) | 0.072 |
| Prior myocardial infarction, n (%) | 71 (24.7) | 133 (34.8) | 0.376 |
| Prior PCI, n (%) | 92 (32.0) | 141 (36.9) | 0.136 |
| Prior CABG, n (%) | 35 (12.2) | 37 (9.7) | 0.143 |
| Atrial fibrillation, n (%) | 39 (13.5) | 45 (11.8) | 0.114 |
| Stroke, n (%) | 11 (3.8) | 10 (2.6) | 0.867 |
| Moderate-severe CKD, n (%) | 42 (14.3) | 78 (20.4) | 0.145 |
| Ischemic DCM, n (%) | 7 (2.4) | 19 (5) | 0.895 |
| Non-ischemic DCM, n (%) | 5 (1.7) | 14 (3.7) | 0.915 |
| Pacemaker, n (%) | 3 (1.1) | 20 (5.2) | 0.418 |
| ICD, n (%) | 2 (0.7) | 5 (1.3) | 0.838 |
| NYHA class II, n (%) | 110 (38.2) | 158 (41.4) | 0.412 |
| NYHA class III, n (%) | 6 (2.1) | 18 (4.7) | 0.412 |
| High risk procedure, n (%) | 158 (55.0) | 204 (53.4) | 0.415 |
| Revised Cardiac Risk index score = 2, n(%) | 143 (49.8) | 137 (35.9) | **0.0004** |
| Revised Cardiac Risk index score ≥ 3, n(%) | 37 (13.0) | 117 (30.6) | 0.335 |
| ASA class III, n (%) | 144 (49.5) | 219 (57.3) | **< 0.001** |
| ASA class IV, n (%) | 1 (0.3) | 19 (4.9) | **0.0003** |
| **Symptoms** | | | |
| Typical angina, n (%) | 22 (7.7) | 40 (10.5) | **< 0.001** |
| Atypical angina, n (%) | 70 (24.0) | 126 (27.7) | 0.710 |
| Dyspnea, n (%) | 118 (41.1) | 166 (43.5) | 0.828 |
| No symptoms, n (%) | 103 (35.9) | 135 (35.3) | 0.345 |
| **Medical Therapy** | | | |
| ACEi/ARBs, n (%) | 190 (66.2) | 268 (70.2) | 0.276 |
| Beta-blockers, n (%) | 167 (58.1) | 247 (64.7) | 0.088 |
| Statins, n (%) | 160 (55.7) | 257 (67.3) | **0.002** |
| Antiplatelet, n (%) | 169 (58.9) | 281 (73.6) | **< 0.001** |
| Oral anticoagulant, n (%) | 53 (18.5) | 93 (24.3) | 0.068 |
| **Laboratory tests** | | | |
| Creatinine, mg/dL | 0.9 (0.78 – 1.07) | 0.93 (0.77 – 1.1) | < 0.181 |
| Hemoglobin, g/dl | 13.7 (12.5 – 14.8) | 13.3 (11.7 – 14.4) | **< 0.001** |
| Platelets, x 1000 | 235 (196 – 300) | 213 (175 – 263) | **0.002** |
| AST, IU/L | 20 (17 – 23.5) | 18 (15 – 23) | **0.001** |
| ALP, IU/L | 18 (14 – 24) | 15 (14 – 20) | **< 0.001** |
| **Echocardiographic parameters** | | | |
| LVEDV, ml | 95 (85 – 119) | 97 (86 – 116) | 0.415 |
| LVEF, % | 55 (52 – 58) | 55 (50 – 55) | 0.067 |
| Diastolic dysfunction grade II, n (%) | 49 (17) | 124 (32.5) | **< 0.001** |
| Diastolic dysfunction grade III, n(%) | 3 (1.0) | 4 (1.0) | 1.000 |
| TAPSE, mm | 24 (21 – 26) | 24 (20 – 27) | 0.875 |
| SPAP, mmHg | 30 (25 – 30) | 30 (26 – 30) | **0.023** |

| **Supplemental table 2** | **Univariable** | | **Multivariable** | |
| --- | --- | --- | --- | --- |
|  | **OR**  **(95%CI)** | **p** | **OR**  **(95%CI)** | **p** |
| Age | 1.01 (0.99 – 1.03) | 0.112 |  |  |
| Male | 1.92 (1.19 – 3.09) | **0.007** | 1.74 (1.09 – 2.79) | **0.020** |
| Familiar History of CAD | 0.88 (0.50 – 1.54) | 0.662 |  |  |
| Hypertension | 9.79 (3.053 – 31.40) | **0.001** | 5.72 (1.70 – 19.15) | **0.005** |
| Dyslipidemia | 1.89 (1.13 – 3.16) | **0.014** | n.s. |  |
| Diabetes | 1.95 (1.27 – 3.01) | **0.002** | n.s. |  |
| Current or former Smoking | 1.01 (0.79 – 1.29) | 0.917 |  |  |
| Coronary artery disease | 4.61 (2.83 – 7.51) | **0.001** | 4.01 (2.30 -6.99) | **< 0.001** |
| Prior myocardial infarction | 2.39 (1.55 – 3.68) | **0.001** |  |  |
| Prior PCI | 4.08 (2.62 – 6.35) | **0.001** |  |  |
| Prior CABG | 1.72 (0.94 – 3.13) | 0.077 |  |  |
| Atrial fibrillation | 1.04 (0.73 – 1.49) | 0.795 |  |  |
| Stroke | 0.61 (0.31 – 1.19) | 0.154 |  |  |
| Moderate-severe CKD | 2.24 (1.38 – 3.63) | **0.001** | 1.74 (1.06 – 2.84) | **0.026** |
| Ischemic DCM | 0.45 (0.10-1.96) | 0.294 |  |  |
| NYHA class | 1.08 (0.75 – 1.57) | 0.662 |  |  |
| High risk procedure | 0.84 (0.55 – 1.28) | 0.429 |  |  |
| Revised Cardiac Risk index score | 1.99 (1.49 – 2.67) | **0.001** | n.s. |  |
| ASA class | 1.08 (0.78 – 1.47) | 0.602 |  |  |
| Symptoms typical angina | 1.09 (0.53 – 2.22) | 0.812 |  |  |
| Symptoms atypical angina | 0.74 (0.45 – 1.24) | 0.263 |  |  |
| Symptoms dyspnea | 0.95 (0.62 – 1.47) | 0.848 |  |  |
| No symptoms | 1.16 (0.75 – 1.80) | 0.489 |  |  |
| LVEF | 1.00 (0.97 – 1.04) | 0.630 |  |  |
| Diastolic dysfunction II,III | 1.86 (1.31 – 2.64) | **0.001** | 1.85 (1.30 – 2.64) | **0.001** |
| Predicted METS | 0.92 (0.50 – 1.72) | 0.817 |  |  |
| ACEi/ARBs | 2.05 (1.22 – 3.44) | **0.007** |  |  |
| Beta-blockers | 3.56 (2.06 – 6.15) | **0.001** |  |  |
| Statins | 4.91 (2.68 – 9.01) | **0.001** |  |  |
| Antiplatelet | 3.21 (1.80 – 5.70) | **0.001** |  |  |
| Oral anticoagulant | 0.80 (0.47 – 1.37) | 0.427 |  |  |

| **Supplemental table 3** | **Univariable** | | **Multivariable** | |
| --- | --- | --- | --- | --- |
|  | **OR**  **(95%CI)** | **p** | **OR**  **(95%CI)** | **p** |
| Age | 0.99 (0.96 – 1.02) | 0.989 |  |  |
| Male | 1.43 (0.72 – 2.86) | 0.300 |  |  |
| Familiar History of CAD | 0.95 (0.41 – 2.19) | 0.922 |  |  |
| Hypertension | 1.06 (0.46 – 2.43) | 0.882 |  |  |
| Dyslipidemia | 1.74 (0.79 – 3.82) | 0.163 |  |  |
| Diabetes | 0.80 (0.39 – 1.62) | 0.545 |  |  |
| Current or former Smoking | 1.27 (0.87 – 1.84) | 0.205 |  |  |
| Coronary artery disease | 2.34 (1.21 – 4.55) | **0.011** | 2.33 (1.11 – 4.88) | **0.025** |
| Prior myocardial infarction | 1.70 (0.89 – 3.25) | 0.107 |  |  |
| Prior PCI | 1.29 (0.67 - 2.48) | 0.444 |  |  |
| Prior CABG | 2.54 (1.14 – 5.63) | 0.022 |  |  |
| Atrial fibrillation | 1.06 (0.61 – 1.85) | 0.827 |  |  |
| Stroke | 0.77 (0.31 – 1.91) | 0.582 |  |  |
| Moderate-severe CKD | 2.05 (1.00 – 4.18) | **0.047** | n.s. |  |
| Ischemic DCM | 0.63 (0.08 – 4.67) | 0.653 |  |  |
| Non-ischemic DCM | 0.88 (0.11 – 6.55) | 0.901 |  |  |
| NYHA class | 1.91 (1.15 – 3.15) | **0.011** | n.s. |  |
| High risk procedure | 1.14 (0.60 – 2.15) | 0.684 |  |  |
| Revised Cardiac Risk index score | 1.52 (1.01 – 2.31) | **0.045** | n.s. |  |
| ASA class | 1.24 (0.79 – 1.92) | 0.338 |  |  |
| Symptoms atypical angina | 0.81 (0.38 – 1.73) | 0.596 |  |  |
| Symptoms dyspnea | 1.42 ( 0.75 – 2.68) | 0.279 |  |  |
| No symptoms | 1.06 (0.55 – 2.05) | 0.840 |  |  |
| LVEF | 0.98 (0.94 – 1.03) | 0.571 |  |  |
| Diastolic dysfunction | 1.36 (0.82 – 2.23) | 0.224 |  |  |
| Predicted METS | 0.21 (1.11 – 0.41) | **< 0.001** | 0.26 (0.12 – 0.55) | **0.001** |
| ACEi/ARBs, n (%) | 0.62 (0.33 – 1.19) | 0.156 |  |  |
| Beta-blockers, n (%) | 1.44 (0.72 – 2.87) | 0.292 |  |  |
| Statins, n (%) | 1.01 (0.52 – 1.94) | 0.971 |  |  |
| Antiplatelet, n (%) | 1.09 (0.560 – 2.15) | 0.784 |  |  |
| Oral anticoagulant, n (%) | 1.99 (1.00 – 3.89) | **0.048** | n.s. |  |
| Stress test strategy | 0.34 (0.15 – 0.76) | **0.008** | 0.33 (0.15 – 0.76) | **0.009** |
| Presence of Ischemia | 0.79 (0.18 – 3.33) | 0.752 |  |  |
